# Supplementary material for: Functional Impact of Neuro-Vascular Bundle Preservation in High Risk Prostate Cancer without Compromising Oncological Outcomes: A Propensity-Modelled Analysis
Source: Cancers (Basel). 2023 Dec 14;15(24):5839. doi: 10.3390/cancers15245839 (PMC10741934; doi:10.3390/cancers15245839)
Supplement: Supplementary file 1 [file cancers-15-05839-s001.zip › cancers-2676831-supplementary.pdf]

Supplemental Material

Supplemental Table S1a: Functional outcomes in patients with uni- vs. bilateral NS-RP before and after IPTW

	Before IPTW				*After IPTW*			
	**Unilateral NS**	**Bilateral NS**	**diff**	***P*-value**	**Unilateral NS**	**Bilateral NS**	**diff**	***P*-value**
Number of patients	516	666			516	666		
Complete EFR, n (%)								
3 months	13 (3)	46 (7)	-0.210	0.001	14 (3)	46 (7)	-0.196	0.003
6 months	36 (7)	85 (13)	-0.197	0.001	36 (7)	83 (13)	-0.186	0.003
12 months	54 (10)	134 (20)	-0.271	<0.001	53 (10)	129 (19)	-0.258	<0.001
24 months	70 (14)	179 (27)	-0.334	<0.001	68 (13)	172 (26)	-0.324	<0.001
EFR, n (%)								
3 months	53 (10)	141 (21)	-0.302	<0.001	52 (10)	137 (21)	-0.292	<0.001
6 months	113 (22)	247 (37)	-0.336	<0.001	115 (22)	242 (36)	-0.314	<0.001
12 months	188 (36)	344 (52)	-0.310	<0.001	193 (37)	334 (50)	-0.261	<0.001
24 months	207 (40)	378 (57)	-0.339	<0.001	209 (40)	367 (55)	-0.296	<0.001
Continence, n (%)								
3 months	430 (83)	572 (86)	-0.074	0.210	431 (84)	572 (86)	-0.064	0.287
6 months	462 (89)	619 (93)	-0.120	0.044	462 (90)	618 (93)	-0.116	0.057
12 months	489 (95)	638 (96)	-0.045	0.459	490 (95)	637 (96)	-0.033	0.601
24 months	501 (97)	646 (97)	0.008	0.896	502 (97)	645 (97)	0.022	0.741
Aid, n (%)								
3 months				<0.001				<0.001
no aid	19 (4)	53 (8)	0.185		19 (4)	53 (8)	0.182	
oral PDE-5 inhibitors	24 (5)	76 (11)	0.248		24 (5)	74 (11)	0.243	
non-oral (MUSE+ISI)	17 (3)	18 (3)	-0.034		16 (3)	17 (3)	-0.033	
erectile dysfunction	456 (88)	519 (78)	-0.281		457 (89)	522 (78)	-0.278	
6 months				<0.001				<0.001
no aid	40 (8)	90 (14)	0.190		39 (8)	89 (13)	0.187	
oral PDE-5 inhibitors	40 (8)	118 (18)	0.303		41 (8)	114 (17)	0.283	
non-oral (MUSE+ISI)	39 (7)	45 (7)	-0.029		39 (8)	45 (7)	-0.028	
erectile dysfunction	398 (77)	413 (62)	-0.332		397 (77)	417 (63)	-0.315	
12 months				<0.001				<0.001
no aid	56 (11)	135 (20)	0.263		54 (11)	131 (20)	0.256	
oral PDE-5 inhibitors	63 (12)	150 (22)	0.274		66 (13)	146 (22)	0.241	
non-oral (MUSE+ISI)	72 (14)	63 (9)	-0.144		75 (15)	62 (9)	-0.160	
erectile dysfunction	325 (63)	319 (48)	-0.307		320 (62)	327 (49)	-0.263	
24 months				<0.001				<0.001
no aid	70 (14)	182 (27)	0.342		68 (13)	175 (26)	0.335	
oral PDE-5 inhibitors	59 (12)	134 (20)	0.238		62 (12)	129 (19)	0.204	
non-oral (MUSE+ISI)	78 (15)	65 (10)	-0.165		80 (16)	65 (10)	-0.175	
erectile dysfunction	308 (60)	286 (43)	-0.341		306 (59)	296 (44)	-0.298	

NS-RP, nerve sparing radical prostatectomy; IPTW, EFR, erectile function recovery; PDE-5, Phosphodiesterase 5: MUSE, medicated urethral system for erection: ISI, intracavernous self-injection with prostaglandin

Supplemental Table S1b: Functional outcomes in all 1340 patients undergoing no vs. uni- vs. bilateral RP for prostate cancer

	**No NS**	**Unilateral NS**	**Bilateral NS**	***P*-value**
Number of patients	158	516	666	
Complete EFR, n (%)				
3 months	1 (0.63)	13 (2.5)	46 (7.0)	<0.001
6 months	1 (0.64)	35 (6.9)	83 (13)	<0.001
12 months	1 (0.67)	52 (10)	124 (20)	<0.001
24 months	3 (2.2)	64 (14)	155 (27)	<0.001
EFR, n (%)				
3 months	6 (3.8)	53 (10)	140 (21)	<0.001
6 months	18 (11)	111 (22)	241 (37)	<0.001
12 months	25 (17)	182 (36)	319 (52)	<0.001
24 months	25 (19)	188 (40)	327 (57)	<0.001
Continence, n (%)				
3 months	99 (65)	423 (83)	569 (86)	<0.001
6 months	110 (74)	443 (89)	602 (93)	<0.001
12 months	114 (82)	454 (95)	586 (96)	<0.001
24 months	104 (84)	432 (97)	540 (97)	<0.001
Aid, n (%)				<0.001
3 months				
no aid	2 (1.3)	13 (2.5)	49 (7.4)	
oral PDE-5 inhibitors	2 (1.3)	24 (4.7)	75 (11)	
non-oral (MUSE+ISI)	2 (1.3)	17 (3.3)	18 (2.7)	
erectile dysfunction	152 (96)	460 (89)	519 (79)	
6 months				<0.001
no aid	4 (2.5)	39 (7.7)	89 (14)	
oral PDE-5 inhibitors	10 (6.4)	39 (7.7)	114 (18)	
non-oral (MUSE+ISI)	7 (4.5)	38 (7.5)	44 (6.8)	
erectile dysfunction	136 (87)	391 (77)	404 (62)	
12 months				<0.001
no aid	4 (2.5)	54 (10)	125 (19)	
oral PDE-5 inhibitors	9 (5.7)	61 (12)	139 (21)	
non-oral (MUSE+ISI)	15 (9.5)	70 (14)	58 (8.7)	
erectile dysfunction	122 (77)	315 (61)	296 (44)	
24 months				<0.001
no aid	6 (3.8)	64 (12)	157 (24)	
oral PDE-5 inhibitors	10 (6.3)	54 (10)	116 (17)	
non-oral (MUSE+ISI)	13 (8.2)	71 (14)	56 (8.4)	
erectile dysfunction	106 (67)	280 (54)	247 (37)	

NS-RP, nerve sparing radical prostatectomy; EFR, erectile function recovery; PDE-5, Phosphodiesterase 5: MUSE, medicated urethral system for erection: ISI, intracavernous self-injection with prostaglandin

Supplemental Table S2: Postoperative complications

Supplemental Table S2a: Morbidity expressed with the Bern CCI after 30d and 90d

	No NS	Unilateral NS	Bilateral NS	*P*-value
Number of patients	158	516	666	
Berne CCI (30 days)*	19 [15 - 22]	22 [15 - 24]	22 [15 - 22]	0.66
Berne CCI (90 days)*	22 [15 - 27]	22 [15 - 34]	22 [15 - 33]	0.67

**only patients who experienced a complication*

Supplemental Table S2b: Numbers of complications according to the Clavien-Dindo Classification (CDC) after 90 days

Clavien-Dindo Grades	No NS	Unilateral NS	Bilateral NS	*P*-value
CDC 1, n (%)	16 (10)	32 (6)	57 (9)	0.17
CDC 2, n (%)	19 (12)	56 (11)	82 (12)	0.73
CDC 3a, n (%)	26 (16)	84 (16)	133 (20)	0.2
CDC 3b, n (%)	2 (1)	14 (3)	17 (3)	0.58
CDC 4a, n (%)	0	3 (1)	3 (0.5)	0.63
CDC 4b, n (%)	0	0	0	-
CDC 5, n (%)	0	0	0	-

CCI, Comprehensive Complication Index; NS, nerve sparing; CDC, Clavien-Dindo Classification

Supplemental Table S2c: Numbers of surgical complications after 90 days in all 1340 patients undergoing pelvic lymph node dissection

	No NS	Unilateral NS	Bilateral NS	*P*-value
Number of patients	158	516	666	
Lymphocele (with and without drainage)	19 (12)	60 (12)	104 (16)	0.12
Lymphocele (with need for drainage)	14 (8.9)	41 (7.9)	76 (11)	0.13
Symptomatic lymphedema	7 (4.4)	10 (1.9)	19 (2.9)	0.21
Neurapraxia (obturator nerve)	8 (5.1)	18 (3.5)	32 (4.8)	0.46
Deep venous thrombosis	0 (0.00)	12 (2.3)	17 (2.6)	0.09
Pulmonary embolism	2 (1.3)	5 (0.97)	7 (1.1)	0.86

Supplemental Table S3: Probability of oncological outcome per treatment group at given time points

Table S3a: All-cause death

Years since RP	***Entire Cohort***	***No NS***	***Uni NS***	***Bi NS***
1	100	100	100	100
2	100 (99 to 100)	100	100 (99 to 100)	100 (99 to 100)
3	99 (99 to 100)	99 (94 to 100)	99 (98 to 100)	99 (98 to 100)
4	99 (98 to 99)	97 (92 to 99)	98 (96 to 99)	99 (98 to 100)
5	97 (96 to 98)	90 (84 to 94)	96 (94 to 98)	99 (98 to 100)
6	97 (95 to 98)	90 (84 to 94)	96 (93 to 97)	99 (98 to 100)
7	96 (95 to 97)	90 (84 to 94)	94 (92 to 96)	99 (98 to 100)
8	96 (94 to 97)	90 (84 to 94)	94 (91 to 96)	98 (97 to 99)
9	95 (94 to 96)	88 (81 to 93)	93 (90 to 95)	98 (97 to 99)
10	94 (92 to 95)	85 (77 to 91)	92 (89 to 95)	98 (96 to 99)
11	94 (92 to 95)	84 (76 to 90)	92 (89 to 94)	98 (95 to 99)
12	92 (90 to 94)	83 (74 to 89)	91 (87 to 93)	97 (94 to 98)

Table S3b: Cancer-specific death

Years since RP	***Entire Cohort***	***No NS***	***Uni NS***	***Bi NS***
1	100	100	100	100
2	100 (99 to 100)	100	100 (99 to 100)	100 (99 to 100)
3	99 (99 to 100)	99 (94 to 100)	99 (98 to 100)	99 (98 to 100)
4	99 (98 to 99)	97 (92 to 99)	98 (96 to 99)	99 (98 to 100)
5	97 (96 to 98)	90 (84 to 94)	96 (94 to 98)	99 (98 to 100)
6	97 (95 to 98)	90 (84 to 94)	96 (93 to 97)	99 (98 to 100)
7	96 (95 to 97)	90 (84 to 94)	94 (92 to 96)	99 (98 to 100)
8	96 (94 to 97)	90 (84 to 94)	94 (91 to 96)	98 (97 to 99)
9	95 (94 to 96)	88 (81 to 93)	93 (90 to 95)	98 (97 to 99)
10	94 (92 to 95)	85 (77 to 91)	92 (89 to 95)	98 (96 to 99)
11	94 (92 to 95)	84 (76 to 90)	92 (89 to 94)	98 (95 to 99)
12	92 (90 to 94)	83 (74 to 89)	91 (87 to 93)	97 (94 to 98)

b

Table S3c: Other cause death

Years since RP	Entire Cohort	No NS	Uni NS	Bi NS
1	100	100	100	100
2	100 (99 to 100)	100	100 (99 to 100)	100 (99 to 100)
3	99 (99 to 100)	99 (94 to 100)	99 (98 to 100)	99 (98 to 100)
4	99 (98 to 99)	97 (92 to 99)	98 (96 to 99)	99 (98 to 100)
5	97 (96 to 98)	90 (84 to 94)	96 (94 to 98)	99 (98 to 100)
6	97 (95 to 98)	90 (84 to 94)	96 (93 to 97)	99 (98 to 100)
7	96 (95 to 97)	90 (84 to 94)	94 (92 to 96)	99 (98 to 100)
8	96 (94 to 97)	90 (84 to 94)	94 (91 to 96)	98 (97 to 99)
9	95 (94 to 96)	88 (81 to 93)	93 (90 to 95)	98 (97 to 99)
10	94 (92 to 95)	85 (77 to 91)	92 (89 to 95)	98 (96 to 99)
11	94 (92 to 95)	84 (76 to 90)	92 (89 to 94)	98 (95 to 99)
12	92 (90 to 94)	83 (74 to 89)	91 (87 to 93)	97 (94 to 98)

Table S3d: Any recurrence

Years since RP	Entire Cohort	No NS	Uni NS	Bi NS
1	100	100	100	100
2	100 (99 to 100)	100	100 (99 to 100)	100 (99 to 100)
3	99 (99 to 100)	99 (94 to 100)	99 (98 to 100)	99 (98 to 100)
4	99 (98 to 99)	97 (92 to 99)	98 (96 to 99)	99 (98 to 100)
5	97 (96 to 98)	90 (84 to 94)	96 (94 to 98)	99 (98 to 100)
6	97 (95 to 98)	90 (84 to 94)	96 (93 to 97)	99 (98 to 100)
7	96 (95 to 97)	90 (84 to 94)	94 (92 to 96)	99 (98 to 100)
8	96 (94 to 97)	90 (84 to 94)	94 (91 to 96)	98 (97 to 99)
9	95 (94 to 96)	88 (81 to 93)	93 (90 to 95)	98 (97 to 99)
10	94 (92 to 95)	85 (77 to 91)	92 (89 to 95)	98 (96 to 99)
11	94 (92 to 95)	84 (76 to 90)	92 (89 to 94)	98 (95 to 99)
12	92 (90 to 94)	83 (74 to 89)	91 (87 to 93)	97 (94 to 98)
